# Effects of Two Anti‐HIV‐1 Regimens on Creatinine and Blood Lipids in Elderly People Living With HIV‐1

**DOI:** 10.1155/arat/6185559

**Published:** 2026-01-06

**Authors:** Peipei Luo, Juan Jin, Jinling Yin, Dingsheng Kong, Haohua Hou, Guoxiang Yang, Huanhuan Ba, Jiajia Li

**Affiliations:** ^1^ Antiviral Clinic, Xi’an Eighth Hospital, Xi’an, China; ^2^ Xi’an Jiaotong University Health Science Center, Xi’an, China, xjtu.edu.cn; ^3^ Department of Surgery, Xi’an Eighth Hospital, Xi’an, China; ^4^ Pharmacy Department, Xi’an Eighth Hospital, Xi’an, China

**Keywords:** creatinine, dyslipidemia, elderly PLWHs, HIV-1

## Abstract

**Background:**

Elderly people living with HIV‐1 (PLWH) are more prone to HIV‐related complications.

**Methods:**

We investigated the creatinine and blood lipids of 153 PLWHs receiving an ANV regimen and 315 PLWHs receiving an EFV regimen.

**Results:**

The results showed that the abnormal rates of creatinine were very low in both groups, and ANV had a lower triglycerides abnormality than EFV. No evident difference in high‐density lipoprotein, low‐density lipoprotein, and total cholesterol was observed between the two groups. The abnormal body mass index of the ANV group aged 66 and above was much less than that of the EFV group.

**Conclusion:**

Both the ANV and EFV regimens did not cause severe kidney damage. ANV had an advantage in controlling dyslipidemia. We strongly recommend elderly PLWHs to choose the ANV regimen.

## 1. Introduction

According to the data released by the United Nations Programme on AIDS, the number of people infected worldwide is close to 40 million. In the past 2 decades, there has been an increase in people living with HIV‐1 (PLWH) among individuals aged 50 and above [1]. At present, the most common is antiretroviral therapy (ART), which helps PLWHs significantly extend their life span. Due to the side effects of ART and the biological effects of HIV infection, PLWHs have a greater incidence rate of comorbidity (such as cardiovascular disease, cancer, diabetes, dyslipidemia, and chronic kidney disease) than the general population [2, 3]. Compared with young people infected with HIV, older PLWHs are at higher risk of comorbidity diseases [4]. More, with the aging population, cardiovascular metabolic diseases and other comorbidities have become important factors in reducing quality of life and mortality in aging PLWHs [5]. The elderly PLWHs are more likely to be affected by various non–HIV‐related acute and chronic comorbidity diseases, including chronic kidney diseases, cardiovascular diseases, diabetes, chronic liver diseases, eye problems, joint pain, degenerative musculoskeletal diseases, and peptic ulcer diseases [6, 7]. The direct side effects of ART are abnormal renal tubular secretion [8, 9] and dyslipidemia [10]. Owing to the increase of risk factors of combined renal injury, such as diabetes and hypertension, in the elderly, the proportion of renal injury has increased [11]. Meanwhile, prevention of obesity‐related factors can reduce weight‐related complications in the elderly PLWH population, including cardiovascular disease, and diabetes [5]. Therefore, in this study, we focused on the effects of two anti‐HIV regimens (EFV regimen, EFV + 3TC + TDF; ANV regimen, ANV/3TC/TDF) on creatinine and dyslipidemia in the elderly PLWHs.

## 2. Methods

### 2.1. Participants Included

We investigated volunteers who received EFV + 3TC + TDF (*n* = 315) or ANV/3TC/TDF (*n* = 153) treatment from January 2022 to July 2024. All individuals signed informed consent forms.

Inclusion criteria are as follows: ① age above 55 years old; ② all PLWHs have been using current antiretroviral drugs for 1 year or more; and ③ the treatment options are EFV + 3TC + TDF or ANV/3TC/TDF.

Exclusion criteria are as follows: ① incomplete baseline data, serum biochemical indicators, etc.; ② the PLWHs changed to another plan during the follow‐up period; ③ other diseases (cirrhosis, chronic kidney disease, malignant tumor, diabetes, hypothyroidism, long‐term hypertension, etc.); ④ merge severe opportunistic infections (tuberculous meningitis, cryptococcal meningitis); and ⑤ heavy smoker and alcoholic.

### 2.2. Procedures

PLWHs take drugs orally once a day: 600 mg EFV + 300 mg 3TC + 300 mg TDF or 150 mg ANV + 300 mg 3TC + 300 mg TDF.

### 2.3. Statistical Analysis

We used SPSS 26.0 statistical software to analyze the data. If the quantitative data did not conform to a normal distribution, the median (25% quartile∼75% quartile) was used to represent it, and the nonparametric Mann–Whitney *U* test was used. If the data follows a normal distribution, it is expressed as mean ± standard deviation, and the difference is evaluated using a *t*‐test. The count data were expressed as frequency and percentage *n* (%), and Pearson’s test was performed *χ* ^2^ inspection. *p* < 0.05 indicates a statistically significant difference.

Abnormality percentage calculation: (1) abnormality percentage of creatinine (%) = number of PLWHs with creatinine < 44 (μmol/L) or > 133(μmol/L)/total number of PLWHs × 100/total number of PLWHs × 100. (2) Abnormality percentage of triglyceride (%) = number of PLWHs with triglycerides > 1.7 mmol/L/total number of PLWHs × 100. (3) Abnormality percentage of high‐density lipoprotein (%) = number of PLWHs with high‐density lipoprotein < 1 mmol/L/total number of PLWHs × 100. (4) Abnormality percentage of low‐density lipoprotein (%) = number of PLWHs with low‐density lipoprotein > 3 mmol/L/total number of PLWHs × 100. (5) Abnormality percentage of total cholesterol (%) = number of PLWHs with total cholesterol > 5.2 mmol/L/total number of PLWHs × 100. (6) Abnormality percentage of body mass index (BMI) (%) = number of PLWHs with BMI > 24 kg/m^2^/total numbe of PLWHs × 100.

## 3. Results

### 3.1. Baseline Characteristics

All baseline demographic and clinical characteristics for both groups, including age, creatinine, triglycerides, total cholesterol, high‐density lipoprotein, low‐density lipoprotein, and BMI, are indiscriminate (Table 1).

**Table 1:** Baseline demographic and clinical characteristics.

	**EFV group (*N* = 315)**	**ANV group (*N* = 153)**	***p* value**

Age (years), median (*μ* ± *δ*)	62.96 ± 7.30	63.26 ± 6.97	0.381
Gender, *n* (%)			0.013
Female	51 (16.1%)	12 (7.8%)	
Male	264 (83.9%)	141 (92.2%)	
TG (mmol/L)	1.59 ± 0.82	1.39 ± 0.79	0.211
TC (mmol/L)	3.91 ± 0.97	3.81 ± 0.81	0.512
LDL (mmol/L)	2.58 ± 0.67	2.61 ± 0.74	0.392
HDL (mmol/L)	1.23 ± 0.49	1.16 ± 0.59	0.215
Creatinine (μmol/L)	66.78 ± 13.12	67.89 ± 13.05	0.650
BMI (kg/m^2^)	22.76 ± 3.24	22.70 ± 2.85	0.878

### 3.2. Abnormality of Creatinine

In the two groups, the creatinine levels of PLWHs under 66 years old were almost 100% normal, and the proportion of abnormal creatinine levels of PLWHs over 66 years old was less than 0.01%.

### 3.3. Abnormality of Triglycerides

**Figure 1:**
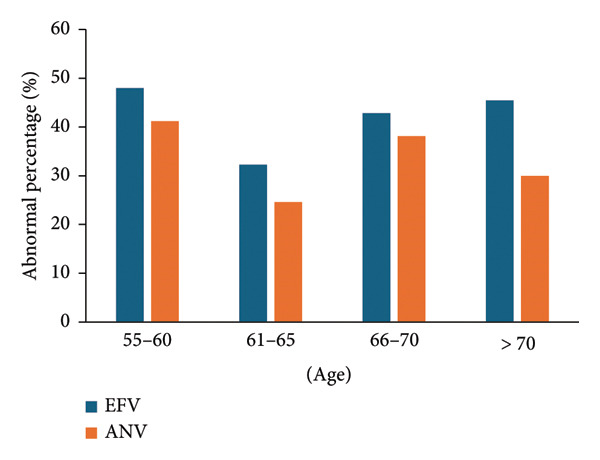
Abnormality of triglycerides in different age groups. More abnormal triglycerides emerged in the EFV group.

The abnormal percentage of triglycerides was lowest in the age of 61–65 and highest in 55–60 (Figure 1). EFV had a higher percentage of anomalies than ANV (*p* = 0.034), with corresponding mean values of 42.1% (42.1%, 95% CI, 32.5%–51.7%) and 33.5% (33.5%, 95% CI, 24.2%–42.8%), respectively.

### 3.4. Abnormality of High‐Density Lipoprotein

**Figure 2:**
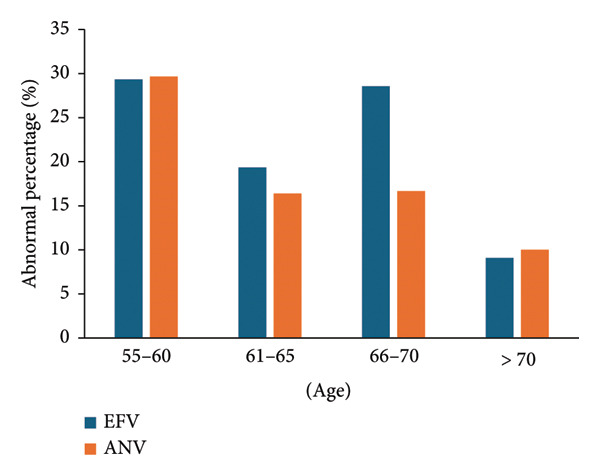
Abnormality of high‐density lipoprotein in different age groups. The abnormality of high‐density lipoprotein showed a decreasing trend with age.

With the increase of age, the abnormality of high‐density lipoprotein in both groups showed a downward trend (Figure 2). No obvious differences were observed between the two groups (*p* = 0.33).

### 3.5. Abnormality of Low‐Density Lipoprotein

**Figure 3:**
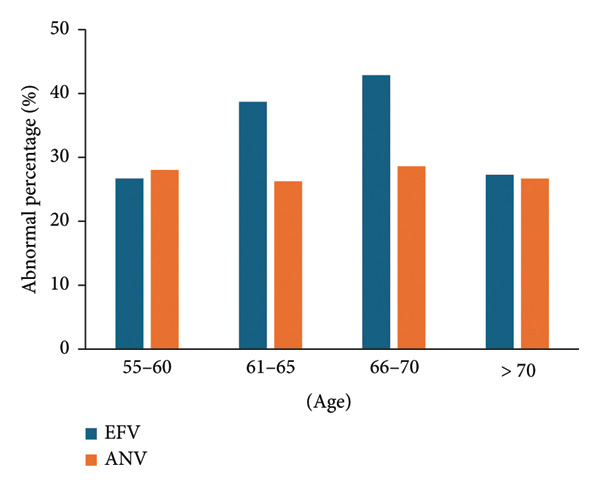
Abnormality of low‐density lipoprotein in different age groups. The abnormality of low‐density lipoprotein in the age group of 61–70 in the EFV group was exacerbated.

The abnormal percentages of low‐density lipoprotein in the EFV group aged 61–65 and 66–70 were 38.7% (38.7%, 95% CI, 29.2%–48.2%) and 42.9% (33.5%, 95% CI, 38%–47.8%), much more than those in other age groups, and no remarkable fluctuations were found in the ANV group (Figure 3). The *p* value for comparing the two groups was 0.203.

### 3.6. Abnormality of Total Cholesterol

**Figure 4:**
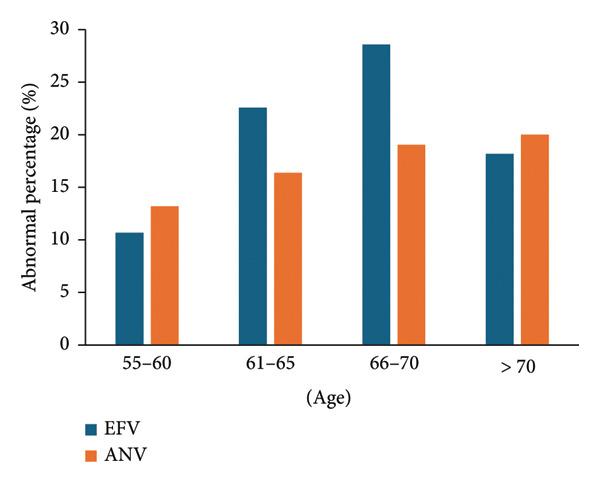
Abnormality of total cholesterol in different age groups.

The ANV regimen led to an upward trend in total cholesterol abnormalities, and EFV had a much higher abnormal percentage in 66–70 compared to other age groups (Figure 4). The overall anomaly percentages of the two groups were 20% (20%, 95% CI, 12.16%–27.84%) (EFV) and 17.2% (17.2%, 95% CI, 13.4%–21%) (ANV), respectively, (*p* = 0.41).

### 3.7. Abnormality of BMI

**Figure 5:**
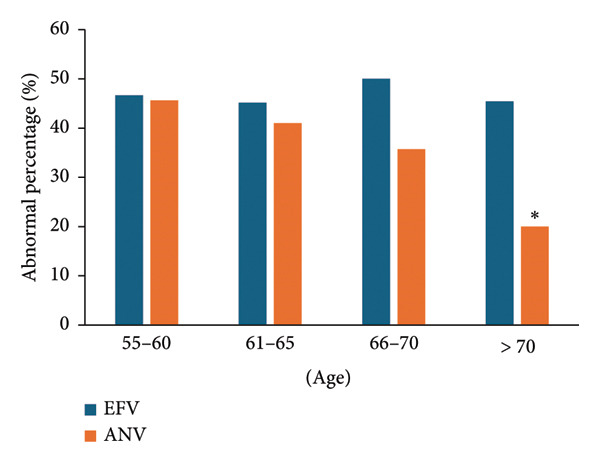
Abnormality of BMI in different age groups. The proportion of abnormal BMI in the ANV group aged 66 and above was lower. ^∗^ *p* < 0.01.

For PLWHs younger than 66 years old, the BMI abnormality percentages of the two groups were very similar (*p* = 0.13) (Figure 5). However, in PLWHs aged 70 and above, the abnormality rate in the ANV group (20.2%) (20.2%, 95% CI, 16.2%–24.2%) was absolutely lower than that in the EFV group (45.5%) (45.5%, 95% CI, 40.5%–50.4%) (*p* < 0.01). In the age group of 66–70, the corresponding values were 35.7% (35.7%, 95% CI, 30.9%–40.5%) and 50.1% (50.1%, 95% CI, 45.1%–55.1%) (*p* < 0.05).

## 4. Discussion

Before ART and during ART, the common complication of HIV‐infected people is impaired renal function, which is related to HIV disease progression, AIDS and non–AIDS‐related events, and increased risk of cardiovascular disease and mortality [12–14]. Our results show that the abnormal rates of creatinine in PLWHs of different age groups were almost zero, indicating that these two treatment options have sufficient safety in terms of renal function. Although complications such as diabetes and hypertension will increase the risk of renal function damage in elderly PLWHs, and the creatinine would also increase physiologically with age [11], theoretically the incidences of these complications are similar in the two groups, so we believe that these effects can be ignored.

One of the known side effects of ART is dyslipidemia [15], including elevated triglycerides, total cholesterol, and low‐density lipoprotein [16, 17], and the reduction of high‐density lipoprotein [18]. In this study, although there was no statistical difference in the abnormal percentages of high‐density lipoprotein, low‐density lipoprotein, and total cholesterol, ANV had a lower triglyceride abnormality rate than EFV (*p* = 0.034) (Figure 1). These suggest that ANV has a slight advantage in controlling lipid abnormalities.

A high BMI has been identified as a signal for kidney injury after receiving ART [19]. The high BMI after ART is also a clue of cardiac metabolic complications such as atherosclerosis and diabetes, which will increase the risk of kidney injury during HIV treatment [20, 21]. We found that there was no discrepancy in the abnormal BMI percentage between the two groups under the age of 65, but the BMI abnormality rates of ANV aged 66–70 and over 70 years old were notably lower than that of EFV, and compared to the EFV’s 48.3%, ANV’s average anomaly rate of 38.6% was still relatively low (Figure 5). Cardiovascular disease is a common and dangerous factor among the elderly, with a 2.3 times higher risk of death for the elderly people receiving ART treatment compared to young people [22]. Therefore, we suggest that PLWHs at risk of cardiac metabolic complications such as atherosclerosis and diabetes should try the ANV regimen.

There are some demerits in our research that are as follows: (1) Lack of data on bone density: both HIV infection and ART may cause a decrease in bone density. (2) Due to the limited number of PLWHs included, this may result in bias in the analysis. (3) It cannot be completely ruled out that some PLWHs may have diabetes or atherosclerosis, which may lead to the decline of glomerular filtration rate. (4) Due to the small number of female PLWHs included, our results cannot reflect the influence on female PLWHs. (5) We cannot absolutely guarantee that the included PLWHs will always receive only one treatment plan, as they may have previously received other drugs. (6) The PLWHs are of a single ethnicity, with the vast majority being Han Chinese.

In conclusion, our research demonstrates that the most notable advantage of the ANV scheme for elderly PLWHs is to reduce the incidence of dyslipidemia, especially for elderly PLWHs aged 66 and above; abnormal BMI is also reduced (which may reduce weight‐related cardiovascular diseases, diabetes, and other complications in the elderly PLWHs), and both schemes have shown excellent performance in terms of renal function safety. Moreover, Ma et al. [23] research shows that compared with EFV+3TC + TDF, ANV/3TC/TDF can better alleviate the side effects of drugs such as dizziness, sadness/depression, tension/anxiety, difficulty in sleeping, and rash/itching, and can also avoid the adverse reactions of EFV to central nervous system and skin problems. Therefore, ANV/3TC/TDF may be a recommended regimen for elderly PLWH who are at risk of atherosclerosis and diabetes.

## Ethics Statement

This study was approved by the Medical Research Ethics Committee of Xi’an Eighth Hospital. All selected patients have signed the ART informed consent form.

## Conflicts of Interest

The authors declare no conflicts of interest.

## Author Contributions

Peipei Luo, Dingsheng Kong, and Juan Jin: conducting research, collecting data, analyzing/interpreting data, designing research, and writing drafts; Jiajia Li and Jinling Yin: collecting data; Haohua Hou, Guoxiang Yang, and Huanhuan Ba: collecting data, analyzing/interpreting data, and conducting clinical follow‐up.

## Funding

This publication was supported by the Xi’an Science and Technology Bureau Innovation Capability Science and Technology Plan Project (21YXYJ0064).

## Data Availability

The data are available and can be requested by sending an email to luopeipei2903@163.com.
